# Development on *Citrus medica* infected with ‘*Candidatus* Liberibacter asiaticus’ has sex-specific and -nonspecific impacts on adult *Diaphorina citri* and its endosymbionts

**DOI:** 10.1371/journal.pone.0239771

**Published:** 2020-10-06

**Authors:** Laurynne C. Coates, Jaclyn Mahoney, John S. Ramsey, EricaRose Warwick, Richard Johnson, Michael J. MacCoss, Stuart B. Krasnoff, Kevin J. Howe, Kathy Moulton, Surya Saha, Lukas A. Mueller, David G. Hall, Robert G. Shatters, Michelle L. Heck, Carolyn M. Slupsky

**Affiliations:** 1 Department of Food Science and Technology, University of California, Davis, California, United States of America; 2 Boyce Thompson Institute for Plant Research, Ithaca, New York, United States of America; 3 Robert W. Holley Center for Agriculture and Health, Emerging Pests and Pathogens Research Unit, USDA Agricultural Research Service, Ithaca, New York, United States of America; 4 Plant Pathology, University of Florida Citrus Research and Education Center, Lake Alfred, Florida, United States of America; 5 Department of Genome Sciences, University of Washington, Seattle, Washington, United States of America; 6 U.S. Horticultural Research Laboratory, Unit of Subtropical Insects and Horticulture, USDA Agricultural Research Service, Fort Pierce, Florida, United States of America; 7 Plant Pathology and Plant-Microbe Biology Section, School of Integrative Plant Science, Cornell University, Ithaca, New York, United States of America; US Department of Agriculture, UNITED STATES

## Abstract

Huanglongbing (HLB) is a deadly, incurable citrus disease putatively caused by the unculturable bacterium, ‘*Candidatus* Liberibacter asiaticus’ (CLas), and transmitted by *Diaphorina citri*. Prior studies suggest *D*. *citri* transmits CLas in a circulative and propagative manner; however, the precise interactions necessary for CLas transmission remain unknown, and the impact of insect sex on *D*. *citri*-CLas interactions is poorly understood despite reports of sex-dependent susceptibilities to CLas. We analyzed the transcriptome, proteome, metabolome, and microbiome of male and female adult *D*. *citri* reared on healthy or CLas-infected *Citrus medica* to determine shared and sex-specific responses of *D*. *citri* and its endosymbionts to CLas exposure. More sex-specific than shared *D*. *citri* responses to CLas were observed, despite there being no difference between males and females in CLas density or relative abundance. CLas exposure altered the abundance of proteins involved in immunity and cellular and oxidative stress in a sex-dependent manner. CLas exposure impacted cuticular proteins and enzymes involved in chitin degradation, as well as energy metabolism and abundance of the endosymbiont ‘*Candidatus* Profftella armatura’ in both sexes similarly. Notably, diaphorin, a toxic Profftella-derived metabolite, was more abundant in both sexes with CLas exposure. The responses reported here resulted from a combination of CLas colonization of *D*. *citri* as well as the effect of CLas infection on *C*. *medica*. Elucidating these impacts on *D*. *citri* and their endosymbionts contributes to our understanding of the HLB pathosystem and identifies the responses potentially critical to limiting or promoting CLas acquisition and propagation in both sexes.

## Introduction

Citrus greening disease, also known as huanglongbing (HLB), is a deadly infectious disease of citrus to which all commercial varieties are susceptible and for which no cure is currently available [1,2]. HLB is characterized by the often slow development of blotchy and asymmetrical yellowing of leaves, vein corking, twig dieback, stunted growth, green and bitter lop-sided fruit, premature fruit drop, and eventually, tree death [1,2]. The putative causative agents of HLB are the uncultured, phloem-limited bacteria: ‘*Candidatus* Liberibacter asiaticus’ (CLas), ‘*Candidatus* Liberibacter americanus’ (CLam), and ‘*Candidatus* Liberibacter africanus’ (CLaf) [1,3]. The Liberibacter species are Gram-negative and members of the Alphaproteobacteria class and *Rhizobiaceae* family [1,4]. In Asia and the U.S.A., CLas is the most prevalent of the three HLB-associated species [1]. It is transmitted in a circulative propagative manner by the phloem-feeding Asian citrus psyllid (*Diaphorina citri*, Hemiptera: Liviidae), and is most efficiently transmitted when acquired by nymphs compared to adults [5–8].

*D*. *citri* were discovered in Florida in 1998 and HLB-symptomatic trees were later discovered in Florida in 2005 [1]. The disease has since devastated the Florida citrus industry with consistent reductions year-after-year in citrus acreage and historically low citrus production [9,10]. The majority of citrus trees in Florida are now infected with CLas [11]. The California citrus industry may be on a similar path, as *D*. *citri* have been detected in most citrus-growing counties and the number of CLas-infected citrus trees continues to rise since CLas was first detected in the state in 2012 [11]. The long period infected plants can remain asymptomatic, and the uneven pathogen distribution make timely identification and removal of diseased trees difficult [12–14]. Limiting HLB spread is further challenged with an insect vector that is developing insecticide resistance [15,16]. Understanding *D*. *citri*-CLas interactions involved in the circulative, propagative transmission of CLas could inform of novel approaches to interrupt and prevent *D*. *citri* transmission of CLas.

CLas infects many organs and tissues of *D*. *citri* including the alimentary canal, hemolymph, hemocytes, muscle tissue, fat tissue, bacteriome, neural tissue, epidermis, reproductive organs, and salivary glands [17–20], and likely has a significant impact on *D*. *citri* health and biology as indicated by a reduction in *D*. *citri* survival and increase in fecundity with CLas infection [21]. Prior studies of CLas-exposed and unexposed *D*. *citri* suggest CLas has significant and systemic effects on *D*. *citri* defense, metabolism, cellular stress, and microbiota [22–34,19], and provide potential responses that may be important in restricting CLas propagation or promoting CLas survival. However, some of the impacts of CLas on *D*. *citri* may be sex-dependent. Analysis of CLas-exposed *D*. *citri* guts, the first barrier to CLas infection, suggests CLas causes oxidative stress in the midguts of adult males, but not females [33]. This oxidative stress could contribute to the greater cellular death that was found in mixed sex gut samples exposed to CLas [32,33]. Furthermore, *D*. *citri* hemolymph (circulatory fluid) exhibits sex-specific abundance of vitellogenin proteins [27]. These vitellogenin proteins might impact CLas infection of the hemolymph because they could have roles in both reproduction and immunity [35].

The major endosymbionts of *D*. *citri*, ‘*Candidatus* Carsonella ruddii’, ‘*Candidatus* Profftella armatura’, and *Wolbachia* [36–39], are differentially abundant in the presence of CLas, depending on *D*. *citri* sex [19,34]. Carsonella and Profftella reside in the bacteriome and are transovarially transmitted to progeny [40]. Based on metagenome analyses, Carsonella is predicted to supply nutritional benefits to *D*. *citri*, particularly in the form of amino acids [36], and Profftella is hypothesized to provide defense through production of a cytotoxic compound, diaphorin [36,41]. Diaphorin is a polyketide which is produced during all *D*. *citri* life stages, is distributed widely in the *D*. *citri* body [42], and may be differentially abundant in response to CLas exposure [24]. Furthermore, CLas may interact with bacteria in the bacteriome, as CLas has been detected in the bacteriome and reproductive organs [18,19] and there is a chance that horizontal gene transfer occurred between Profftella to CLas [43]. *Wolbachia* is systemic in *D*. *citri* [19,44] and while its function is unknown, it sometimes colocalizes in the gut with CLas [26,33], and may also interact with CLas [45]. The differential responses of male and female *D*. *citri* and their endosymbionts to CLas may contribute to the reported, albeit inconsistent, sex differences in susceptibility to CLas [46,47,19,48]. From invertebrates to vertebrates, many animal species display sex-dependent immunity and susceptibility to infectious diseases [49–51]. Yet, despite potential sexual dimorphism in *D*. *citri* immune response and susceptibility to CLas, the underlying differences and similarities between males and females in their molecular responses to CLas have largely been uncharacterized.

Here, we sought to determine the shared and sex-specific responses of adult *D*. *citri* and their endosymbionts to CLas using a systems biology approach based on 16S rRNA gene microbial community analysis, qPCR quantification of the major endosymbionts, polyA transcript-enriched gene expression analysis, LC/MS-based proteomics, and ^1^H-NMR-based metabolomics. Since we performed the extractions for each analysis on the same samples, we directly compared and integrated the results. We found substantial shared and sex-specific differences in response to CLas exposure in the *D*. *citri* microbial community, transcript-level expression of genes, protein abundance, and metabolism. The impacts of CLas on male and female *D*. *citri*, whether a direct result of CLas infection in *D*. *citri* or an indirect result of CLas infection of citrus, are important in understanding the complexities of the HLB pathosystem. Additionally, the responses shared between males and females to CLas exposure are particularly crucial in efforts to understand the mechanisms of CLas infection in *D*. *citri*, and ultimately help with the development of interdiction molecules to efficiently interrupt and prevent CLas transmission in both sexes.

## Materials and methods

### *D*. *citri* colonies

*D*. *citri* were reared in synchrony on healthy or CLas-infected citron (*Citrus medica*), and from each of these colonies 1–5 day old adults were collected, separated by sex, and weighed. *D*. *citri* were reared in growth chambers at 25°C and 70% humidity with a 14-hour light cycle. Ten individual male and ten individual female *D*. *citri* were collected from each of the two colonies for DNA extraction to estimate infection rate and determine, by qPCR, the CLas density in individuals. In addition, five biological replicates consisting of 75 *D*. *citri* each (i.e. each replicate contained a pool of 75-individuals) were collected from each colony and for each sex for sequential extraction of metabolites, DNA, RNA and proteins. *D*. *citri* were stored at -75°C prior to extraction.

### Metabolite, DNA, RNA and protein extraction

DNA was extracted from individual *D*. *citri* reared on citron by grinding with a pestle, adding 150 μL 1X TE buffer and 150 μL phenol, vortexing, and centrifuging at 16,100 x g and 4°C for 12 min. Of the supernatant, 150 μL was combined with 75 μL of 7.5 M ammonium acetate and 450 μL of 95% ethanol, and centrifuged at 16,100 x g and 4°C for 25 min. The supernatant was discarded and the pellet washed with 500 μL of ice cold 70% ethanol, then centrifuged at 16,100 x g and 4°C for 10 min. The supernatant was discarded, the pellet was dried 10–15 min, and the DNA was eluted and transferred in 27 μL nuclease-free water.

Metabolites, DNA, RNA, and proteins were sequentially extracted from each 75-individual pooled sample of *D*. *citri*, using a method adapted from Roume and others [52]. See S1 File for details on extraction protocol.

The purity and quantity of DNA was estimated using gel electrophoresis, Nanodrop 2000c (Thermo Scientific) and Qubit 3.0 fluorometer with Qubit dsDNA HS assay kit (Invitrogen). The purity and quantity of RNA was assessed with the Nanodrop, Qubit 3.0 fluorometer with RNA HS assay kit, and the Bioanalyzer with the RNA 6000 Nano kit (Agilent). The Qubit dsDNA HS assay kit was also used to confirm that there were minimal amounts DNA contaminating the RNA extracts. RNA and DNA were stored at -75°C.

### Bacterial density

The densities of CLas and the *D*. *citri* endosymbionts Carsonella, Profftella, and *Wolbachia* were determined using quantitative PCR. *16S rRNA* specific for Liberibacter was amplified using the primer set USHRL-CL1f and USHRL-CL1r [53], Carsonella *16S rRNA* was amplified with primers Myc-F and Myc-R, Profftella *16S rRNA* was amplified with primers Syn-F and Syn-R and *Wolbachia ftsZ* was amplified with primers ftsZ-F and ftsZ-R [37]. *D*. *citri Rps20* was used as an internal reference for *D*. *citri* genome copy number, and was amplified by qPCR using primers Dci-S20-L and Dci-S20-R [53] to calculate density of the endosymbionts or CLas. For details on conditions of quantitative polymerase chain reaction and thermal cycler conditions, see S1 File.

CLas density was compared between individual CLas-infected males and individual CLas-infected females, as well as between 75-individual pooled CLas-exposed male samples and 75-individual pooled CLas-exposed female samples, using the two-tailed t-test in GraphPad Prism version 6.05 (La Jolla, CA, USA). Endosymbiont densities in the 75-individual pooled samples were log_10_-transformed, and compared between control males and CLas-exposed males, and between control females and CLas-exposed females using unpaired, two-tailed t-test. To control the number of false discoveries arising from multiple t-tests, p-values were used in R p.adjust (stats package version 3.4.3) to determine the Benjamini-Hochberg FDR, and only the discoveries meeting an FDR of 5% were interpreted as statistically significant. Using GraphPad Prism, linear relationships (Pearson correlation) between log_10_-transformed densities of CLas and other endosymbionts were investigated in the 75-individual pooled CLas-exposed male samples and 75-individual pooled CLas-exposed female samples.

### Microbial community

The V4 region of the 16S rRNA gene was amplified in the DNA extracted from 75-individual pooled *D*. *citri* samples using primers F515 and R806, altered as described by Bokulich and others [54]. The forward primer contained an adapter, linker, and barcode with a unique eight-base-sequence for each sample. See S1 File for details on PCR conditions and purification steps followed in preparation of amplicons. The UC Davis DNA Technologies Core assessed the amplicon library using a Bioanalyzer and an HS DNA assay, and found minor amounts of primer-dimers. The primer-dimers were removed with KAPA Pure Beads, and the library was subsequently sequenced on the Illumina MiSeq platform with V2 chemistry to generate paired end 250 reads. Reads containing ≥ 75% bases with greater than Q30 quality score were retained.

Reads were trimmed with trimmomatic version 0.36 [55], further processed and analyzed using QIIME version 1.9.1 [56], and aligned to the SILVA ribosomal RNA gene database [57] using SortMeRNA clustering and closed-reference OTU picking [58]. Median relative abundances of the three most abundant genera were compared between control males and CLas-exposed males, and control females and CLas-exposed females with Mann-Whitney U two-tailed test using GraphPad Prism. The median relative abundance of Liberibacter was also compared between CLas-exposed males and CLas-exposed females with Mann-Whitney U two-tailed test. R p.adjust was used to determine the Benjamini-Hochberg false discovery rate for each p-value. False positives resulting from multiple comparisons were controlled by setting an FDR of 5%.

### Diaphorin isolation and ^1^H-NMR spectroscopy analysis

Diaphorin was isolated from *D*. *citri* following methods described by Nakabachi et al [36] and Szebenyi et al [59]. Purified diaphorin was resuspended in 10 mM phosphate buffer and 4.608 mM 3-(Trimethylsilyl)-1-propanesulfonic acid (DSS-d_6_) standard in > 98% v/v D_2_O and 0.2% w/v NaN_3_ was added at 10% volume. Diaphorin was analyzed by ^1^H-NMR spectroscopy at 298 K on a 600 MHz Bruker Avance III Nuclear Magnetic Resonance Spectrometer using the noseypr1d pulse sequence. Chenomx NMR Suite version 8.13 Spin Simulator and Compound Builder was used to determine the multiplicity and J-coupling for protons on diaphorin, and to make a library entry (S1 Table). The resulting diaphorin library entry was used to identify and quantify diaphorin in metabolites extracted from 75-individual pooled *D*. *citri* samples. Annotation of diaphorin ^1^H chemical shifts and coupling constants in water were similar to the ^1^H chemical shifts and coupling constants reported in methanol by Nakabachi et al [36] and Szebenyi et al [59].

### Metabolome analysis

Polar metabolites were dried with a centrifugal vacuum (miVac Duo Concentrator by GeneVac) and resuspended in 260 μL of 10 mM phosphate buffer. The resuspension was centrifuged at 10,000 x g and 4°C for 10 min, then 207 μL was removed and combined with 23 μL of DSS-d6 standard in > 98% v/v D2O and 0.2% w/v NaN_3_. The pH was adjusted with NaOH and HCl to 6.7–6.9 and recorded, and 180 μL of the metabolite solution with standard was placed in a 3 mm nuclear magnetic resonance (NMR) glass tube. Metabolite samples were run on the 600 MHz Bruker Avance III Nuclear Magnetic Resonance Spectrometer following the same parameters used for analysis of diaphorin. Metabolites were identified and quantified using Chenomx NMR Suite version 8.13, and the concentrations were adjusted for dilution.

Mean metabolite concentrations were compared between control males and CLas-exposed males, and between control females and CLas-exposed females, using unpaired, two-tailed t-tests through GraphPad Prism. The resulting p-values were used with R p.adjust and the Benjamini-Hochberg method to control false discoveries resulting from multiple t-tests. Differences in group means were considered statistically significant if the false discovery rate was at or below 5%.

The pathway/genome databases for *D*. *citri* (versions 1.0 and 2.0), CLas strains psy62 (version 19.0) and gxpsy (version 19.0), *C*. *sinensis* (version 4.0) and *C*. *clementina* (version 4.0) available at citrusgreening.org, and pathway/genome databases for *Wolbachia* strain wMel (version 21.5), Profftella strain DC (version 21.5), and Carsonella strain DC (version 21.5) available through the BioCyc database collection, were used for metabolite pathway analyses.

### RNA-seq

RNA was sent to the DNA Technologies and Expression Analysis Core at the University of California, Davis for library preparation and sequencing. Polyadenylated transcripts were selected, reverse-transcribed, and amplified using the KAPA Stranded mRNA-Seq kit to generate a library for each sample. Libraries were assessed with the Bioanalyzer and Agilent DNA 1000 kit and quantified with the Qubit fluorometer and dsDNA HS assay kit (Invitrogen). Equimolar libraries were pooled and sequenced across three lanes on the Illumina HiSeq 4000 platform to generate paired-end 100 base reads. Only reads containing ≥ 80% bases of > Q30 were retained and demultiplexed according to unique library barcodes.

The Amazon Machine Image provided by the UC Davis Bioinformatics Core was used when running the Galaxy graphical user interface. Using this GUI [60], Scythe version [61] was used to remove adapters from reads and reads were trimmed and filtered with Sickle version 1.33 [62]. Paired-end reads were aligned to the *D*. *citri* genome (version 1.1). Cufflinks 2.2.1 was used to convert the genome annotation file version 1.0 into GTF format and HISAT2 2.1.0 was used to extract exons and build genome indices for alignment [63,64]. The SAM files generated from HISAT2 were converted to BAM files and sorted using samtools version 1.3.1 [65]. Sorted BAM files were used to generate gene count tables with StringTie version 1.3.3, and were then modified for downstream analysis in R version 3.4.3 and edgeR version 3.20.9 [66,67]. See S1 File for parameters used with the aforementioned programs.

Using edgeR, libraries were adjusted to the effective library sizes determined by the trimmed mean of M-values method, genes with counts less than one per million and present in fewer than five samples were removed, dispersions were estimated by the Cox-Reid profile-adjust likelihood method, the negative bionomial generalized linear model was fitted, testing of differential expression was performed with the quasi-likelihood F-test, and the false discovery rate was controlled at 5%.

Analyses of function for differentially expressed genes were performed using manually curated genes [68] and the pathway/genome database for *D*. *citri* version 1.0 created with Pathway Tools and MetaCyc by Citrus Greening Solutions—a USDA NIFA project [68]. Annotations were checked using InterProScan and NCBI BLASTp and alignments to *Drosophila*.

### Mass spectrometry-based quantitative proteomics

The method employed by Ramsey et al [24] was followed for protein purification and mass spectrometry analysis. See S1 File for further details on protein purification and mass spectrometry analysis. Pairwise comparisons of spectral counts in biological replicate samples from different sample classes were performed using Fisher’s Exact Test with a Benjamini-Hochberg adjusted p-value cutoff to identify differentially abundant proteins. Annotations were checked using InterProScan and NCBI BLASTp and alignments to *Drosophila*.

## Results and discussion

### CLas exposure has sex-dependent impacts on *D*. *citri* microbial community composition

**Fig 1:**
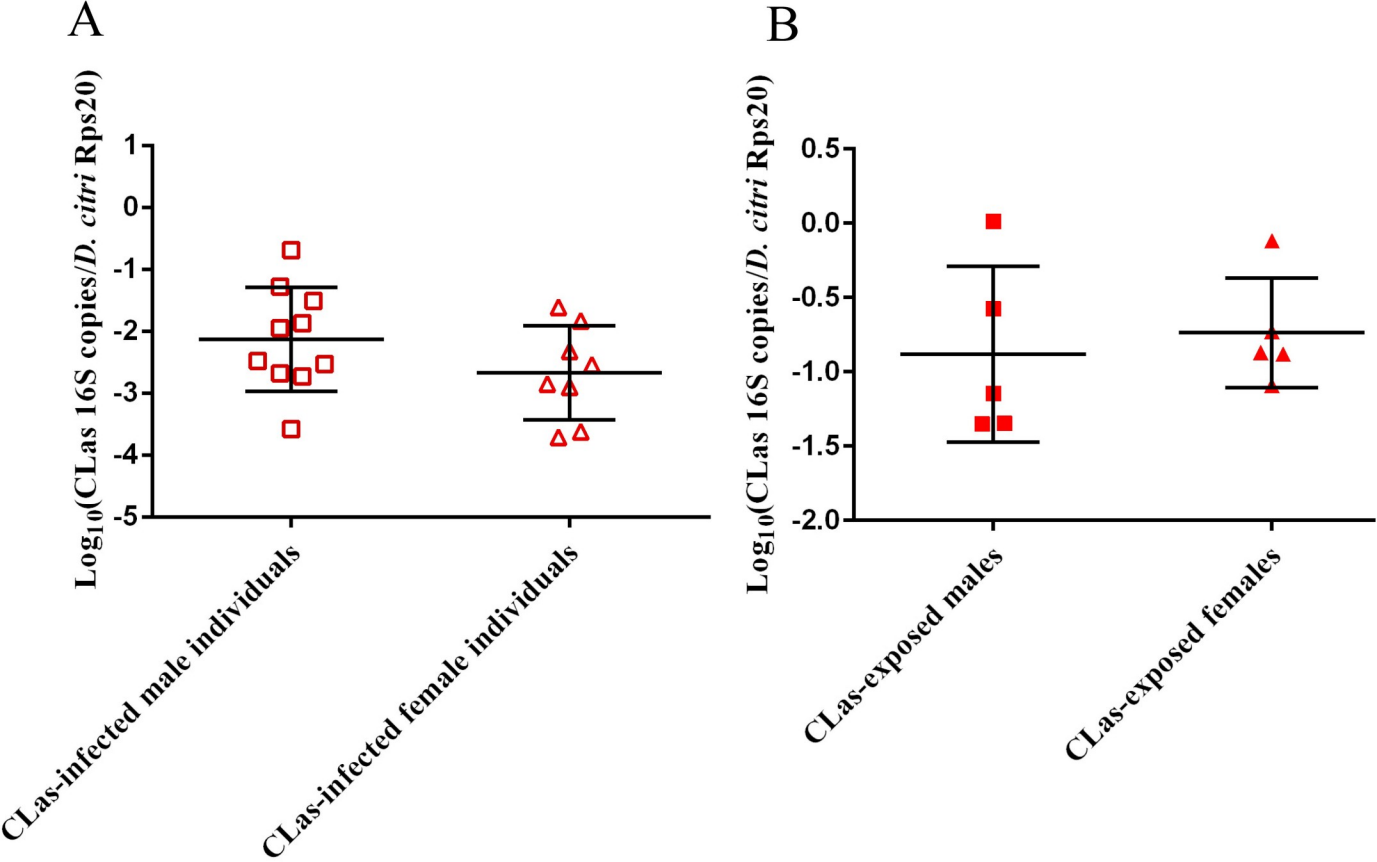
CLas density in FL *D*. *citri* reared on FL CLas-infected *C*. *medica*. (A) The density of CLas in individual and (B) 75-individual pooled *D*. *citri* samples was determined using qPCR. A significant difference between mean densities of CLas in CLas-infected male and female individuals or between 75-individual pooled, CLas-exposed male and female *D*. *citri* was determined using a two-tailed, unpaired t-test.

**Fig 2:**
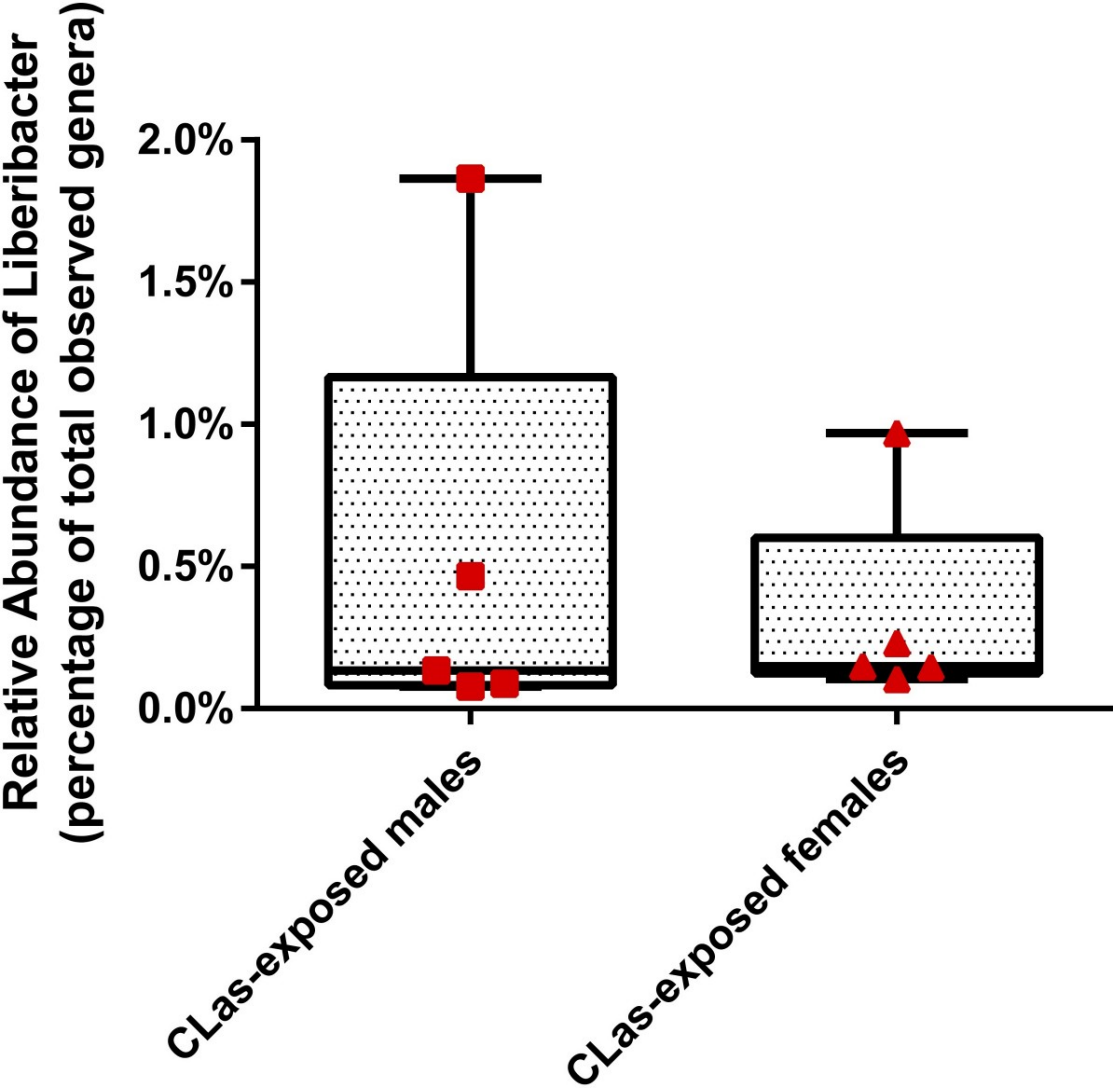
Liberibacter relative abundance in *D*. *citri*. The relative abundance of Liberibacter in 75-individual pooled CLas-exposed males and females was determined using 16S rRNA gene sequencing. A significant difference between median relative abundances of Liberibacter in CLas-exposed males and females was determined with Mann-Whitney U two-tailed test.

Out of the ten male individuals and ten female individuals sampled from the colony of *D*. *citri* reared on CLas-infected *C*. *medica*, ten and eight contained CLas respectively. Of these infected individuals, the CLas density was not significantly different between males and females (Fig 1A). Similarly, in the 75-individual pooled *D*. *citri* samples, the CLas density (Fig 1B) and Liberibacter (presumably CLas) relative abundance at the genus level (Fig 2) were not significantly different between CLas-exposed males and CLas-exposed females. Quantitative PCR targeting CLas was performed on DNA extracts from individual control males, individual control females, 75-individual pooled control males, and 75-individual pooled control females, but no amplification was detected after 40 cycles.

Microbial community analysis of 16S ribosomal RNA gene sequences and relative abundance at the genus level revealed Profftella, Carsonella, and *Wolbachia* to be the most abundant of the bacteria and archaea detected in male and female adult *D*. *citri* (Table 1). Despite a high percentage of males and females carrying Liberibacter (estimated at 80–100%), and a lifetime of CLas exposure, Liberibacter comprised a very small percentage of the *D*. *citri* microbiome (0.08–1.86%) (Fig 2). Interestingly, one CLas-exposed male sample and one CLas-exposed female sample had notably higher CLas relative abundance than the median. These two samples also had the highest CLas densities of their respective conditions (almost 10-fold greater than the mean) (Fig 1B) which likely explains the high CLas relative abundance in these samples. Profftella abundance was lower in CLas-exposed males and females compared to those not exposed (Table 1), which is consistent with lower Profftella relative abundance in CLas-exposed mixed sex adults [38]. However, the relative abundances of Carsonella and *Wolbachia* were greater in CLas-exposed than control adults, with the difference in *Wolbachia* relative abundance between control and CLas-exposed females not statistically significant (Table 1). For all three endosymbionts, the fold difference in relative abundance between control and CLas-exposed *D*. *citri* was approximately the same in males and females.

**Table 1:** Relative abundance of the predominant genera in male and female adults. The median relative abundance (as percentage) was compared between control and CLas-exposed groups for males and females, and significant differences determined using the Mann-Whitney U two-tailed test with an FDR of 5%. The median CLas relative abundance was 0.13% in CLas-exposed males and 0.15% in CLas-exposed females.

	Male					Female				
Genus	Control	CLas-Exposed	Fold Difference	p- value	FDR	Control	CLas-Exposed	Fold Difference	p- value	FDR
Carsonella	11.24%	16.24%	1.444	0.0079	0.0111	24.30%	31.60%	1.301	0.0079	0.0111
Profftella	79.39%	70.57%	0.8889	0.0079	0.0111	68.60%	59.62%	0.8691	0.0079	0.0111
*Wolbachia*	9.306%	12.36%	1.328%	0.0079	0.0111	6.874%	8.383%	1.220	0.1508	0.1759

**Fig 3:**
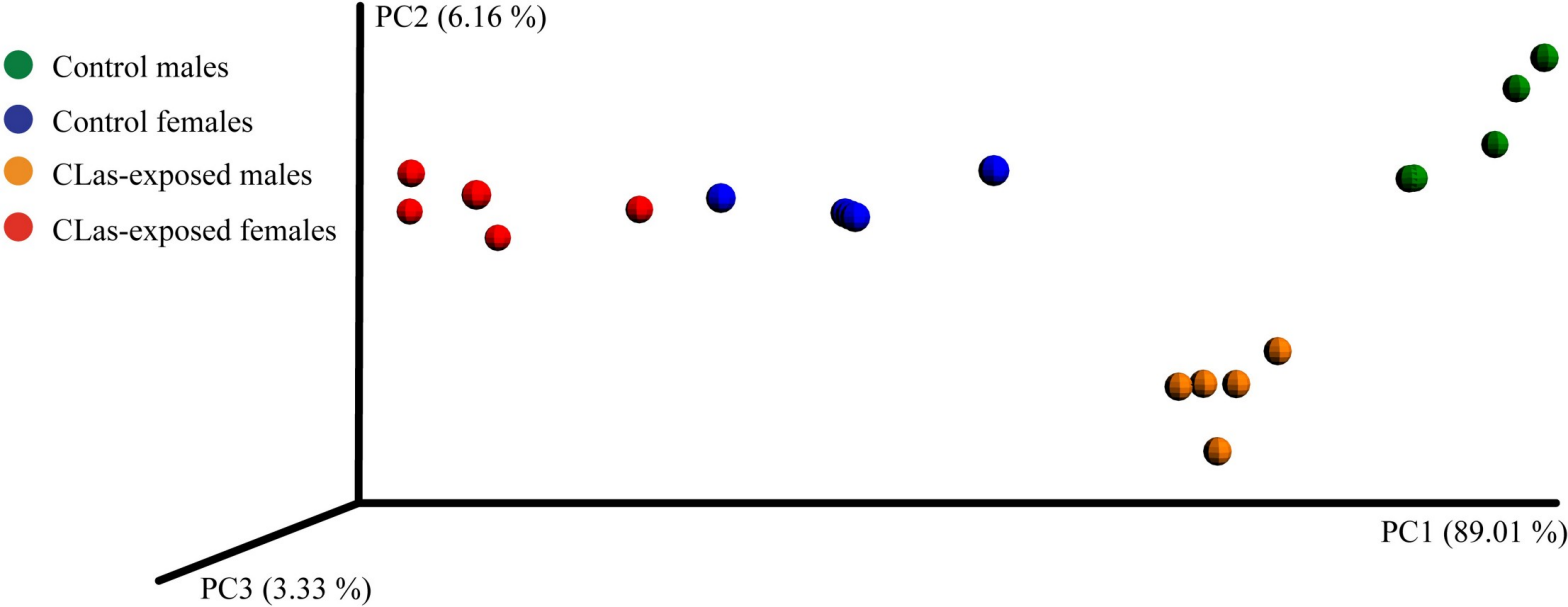
Principal coordinates analysis of weighted UniFrac distances between *D*. *citri* microbial communities based on 16S rRNA gene sequences. Microbial communities of CLas-exposed males are depicted in orange, CLas-exposed females are depicted in red, control males are depicted in green, and control females are depicted in blue.

Principal coordinate analysis of weighted UniFrac distances between *D*. *citri* microbial communities (β-diversity) with even sampling suggest that the differences between male and female microbial communities were greater than the changes in microbial community that occur in response to CLas exposure (Fig 3). The differences between control males and females in microbial community composition and abundance likely contributes to the dissimilarity in microbial communities between CLas-exposed males and females. According to the distance between control males and CLas-exposed males, CLas exposure had a larger impact on the male microbial community than on the female microbial community (Fig 3).

**Fig 4:**
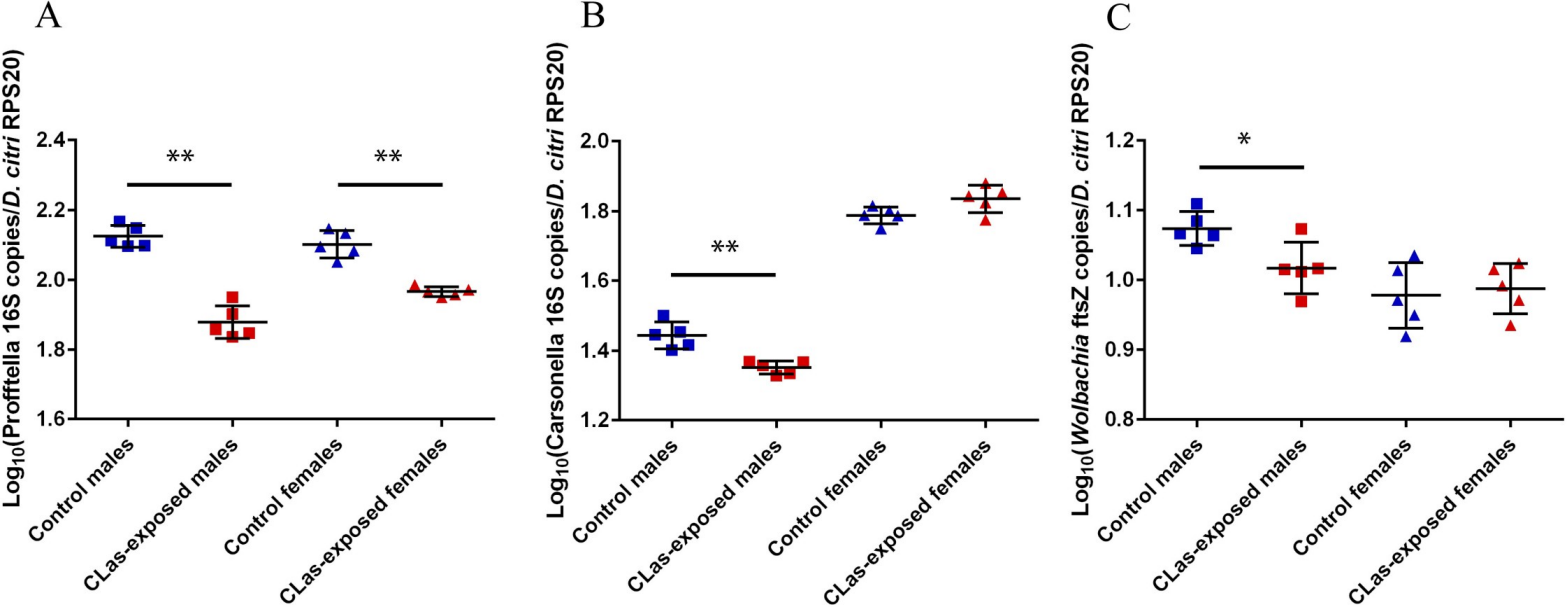
Endosymbiont densities in *D*. *citri*. The density of Profftella (A), Carsonella (B), and *Wolbachia* (C) in adult *D*. *citri* was measured by copies of Profftella 16S rRNA gene, Carsonella 16S rRNA gene, and *Wolbachia* filamentation temperature-sensitive protein Z gene (*ftsZ*), respectively, per *D*. *citri* ribosomal protein S20 gene. Mean log_10_-transformed density of each bacterium in control and CLas-exposed males (blue and red squares, respectively), and in control and CLas-exposed females (blue and red triangles, respectively) was compared using unpaired, two-tailed t-test, with FDR of 5%.

The densities of CLas and the three major *D*. *citri* endosymbionts, Carsonella, Profftella, and *Wolbachia* were determined using quantitative polymerase chain reaction (qPCR). Similar to the relative abundance of Profftella, the density of Profftella was lower in CLas-exposed *D*. *citri* compared to controls (Fig 4A). However, under different experimental conditions, others have found opposite changes in Profftella density with CLas exposure in males versus females [19,34], and this suggests there are other abiotic or biotic factors influencing how CLas exposure impacts the *D*. *citri* sexes. Despite our finding of lower Profftella relative abundance and density in CLas-exposed versus control *D*. *citri*, we did not find the density of Profftella to correlate with the density of CLas in CLas-exposed males or CLas-exposed females (Fig A in S1 Fig, p-value > 0.05).

**Fig 5:**
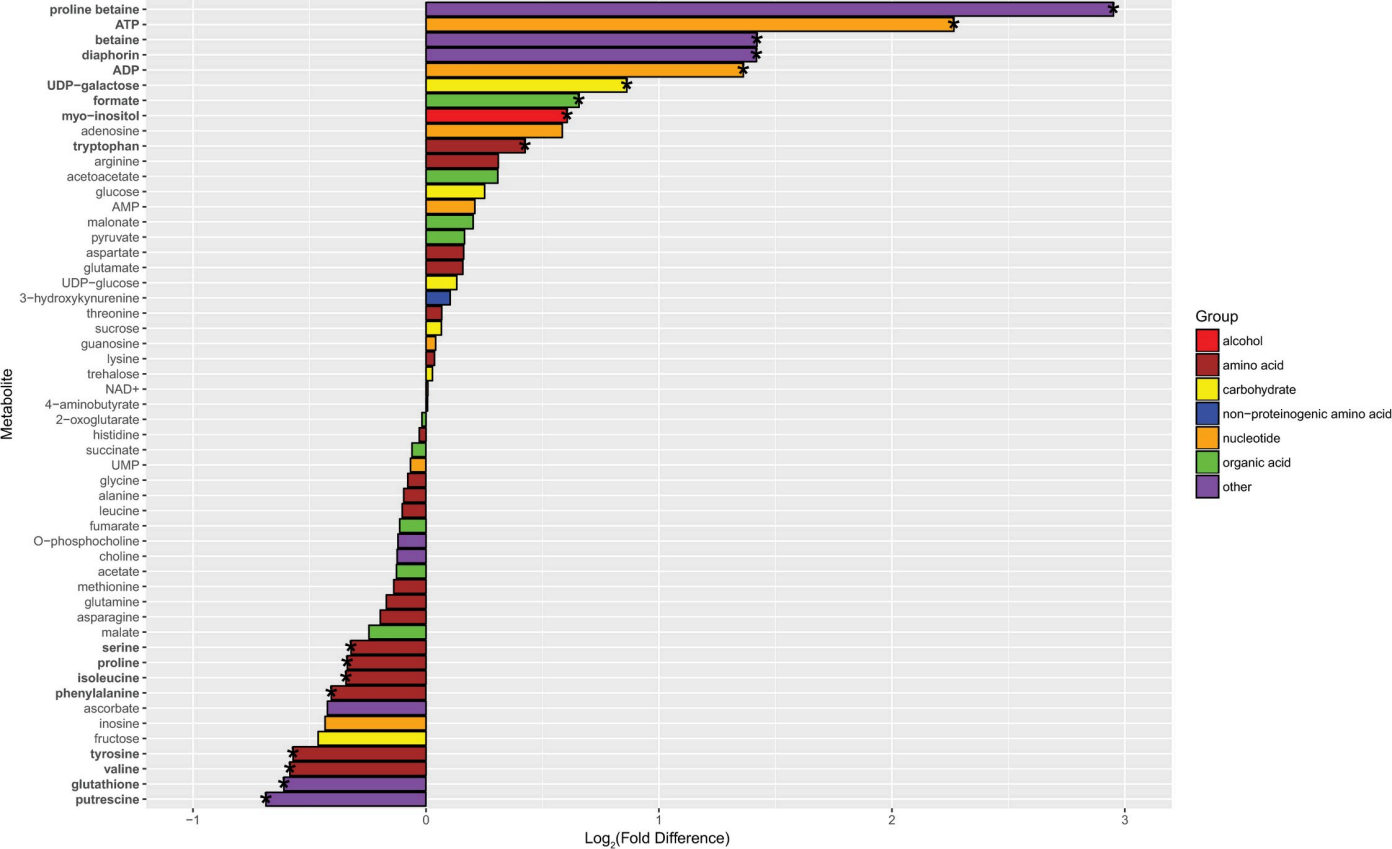
Metabolite log_2_-fold differences between control and CLas-exposed males. Positive fold differences indicate a higher concentration in CLas-exposed males, while negative fold differences indicate a higher concentration in control males. Metabolites in bold and corresponding bars with an asterisk are significantly differentially abundant between control and CLas-exposed males. Metabolites are colored by type of molecule.

**Fig 6:**
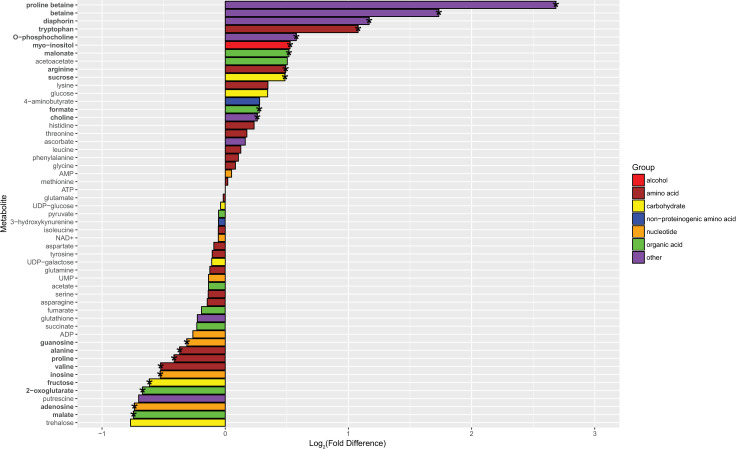
Metabolite log_2_-fold differences between control and CLas-exposed females. Positive fold differences indicate a higher concentration in CLas-exposed females, while negative fold differences indicate a higher concentration in control females. Metabolites in bold and corresponding bars with an asterisk are significantly differentially abundant between control and CLas-exposed females. Metabolites are colored by type of molecule.

We also quantified a polyketide produced by Profftella, called diaphorin, which is toxic to mammalian, insect, and yeast cells [36,41]. It is also structurally similar to the polyketide, pederin [43] which appears to deter predators of the host *Paederus* beetle, and is reported to have antibacterial activity against *Mycobacterium phlei*, and minimal antibacterial activity to *Bacillus subtilis*, and *Escherichia coli* [69]. Although there are no reports of diaphorin’s effects on bacteria, Ramsey et al found altered abundance of Profftella polyketide synthase proteins and a greater ratio of diaphorin to a diaphorin-related polyketide with CLas exposure, and this raises the possibility that diaphorin production is responsive to CLas infection in adult *D*. *citri* [24]. However, it remains to be determined if diaphorin serves as a defensive compound to predators or microbial pathogens of *D*. *citri*. We found the concentration of diaphorin was, on average, two times higher in CLas-exposed males and CLas-exposed females compared to controls (Figs 5 and 6, S1 Dataset). Since Profftella density was concurrently lower in CLas-exposed *D*. *citri* compared to controls, this finding indicates that Profftella increased diaphorin production in response to CLas exposure. Yet diaphorin production might not be directly responsive to CLas density, as we did not find a correlation between diaphorin concentration and CLas density in CLas-exposed *D*. *citri* (S2 Fig). Since diaphorin is toxic to the *D*. *citri* predator, *Harmonia axyridis* [41], its heightened production in CLas-exposed *D*. *citri* could have important implications for predation of *D*. *citri* and thus for spread of CLas. Nonetheless, the high abundance of diaphorin in control and CLas-exposed *D*. *citri* (among the top eight most abundant metabolites quantified here, and similar in abundance to AMP in control *D*. *citri* or proline in CLas-exposed *D*. *citri*) suggests that diaphorin, and thus Profftella, play significant roles in *D*. *citri* biology which warrant further investigation.

Unlike Profftella, the changes in relative abundance of Carsonella and *Wolbachia* were discordant with the changes in density of Carsonella and *Wolbachia*. While the relative abundance of Carsonella was higher with CLas exposure in both sexes (Table 1), the density of Carsonella was lower with CLas exposure in males only (Fig 4B). Similarly, *Wolbachia* relative abundance was greater in CLas-exposed males than controls (Table 1), but *Wolbachia* density was lower in CLas-exposed males (Fig 4C). Even though Carsonella and *Wolbachia* densities were lower in CLas-exposed males versus controls, comparatively Profftella was much lower in CLas-exposed males versus controls. The greater difference in Profftella density between CLas-exposed and control males may explain why Profftella relative abundance was lower, while Carsonella and *Wolbachia* relative abundances were higher in CLas-exposed males versus controls. Neither Carsonella density (Fig B in S1 Fig) nor *Wolbachia* density (Fig C in S1 Fig) correlated with CLas density for CLas-exposed males. As such, the impact of CLas exposure on these endosymbionts may not depend on CLas density. The changes in male *Wolbachia* and Carsonella densities support prior findings of lower Carsonella density in lab-reared, CLas-exposed males [19], and lower Carsonella and *Wolbachia* densities in field-collected, CLas-exposed males [34]. However, the sex-specific manner in which CLas exposure impacted *D*. *citri* endosymbiont densities in this study is not always consistent with differing citrus hosts [19] or environmental conditions [34]. Therefore, certain abiotic or biotic factors are likely influencing the sex-specific responses to CLas exposure and must be considered prior to analyzing males and females together.

### CLas exposure is associated with major sex-specific changes in the *D*. *citri* transcriptome, proteome, and metabolome

**Fig 7:**
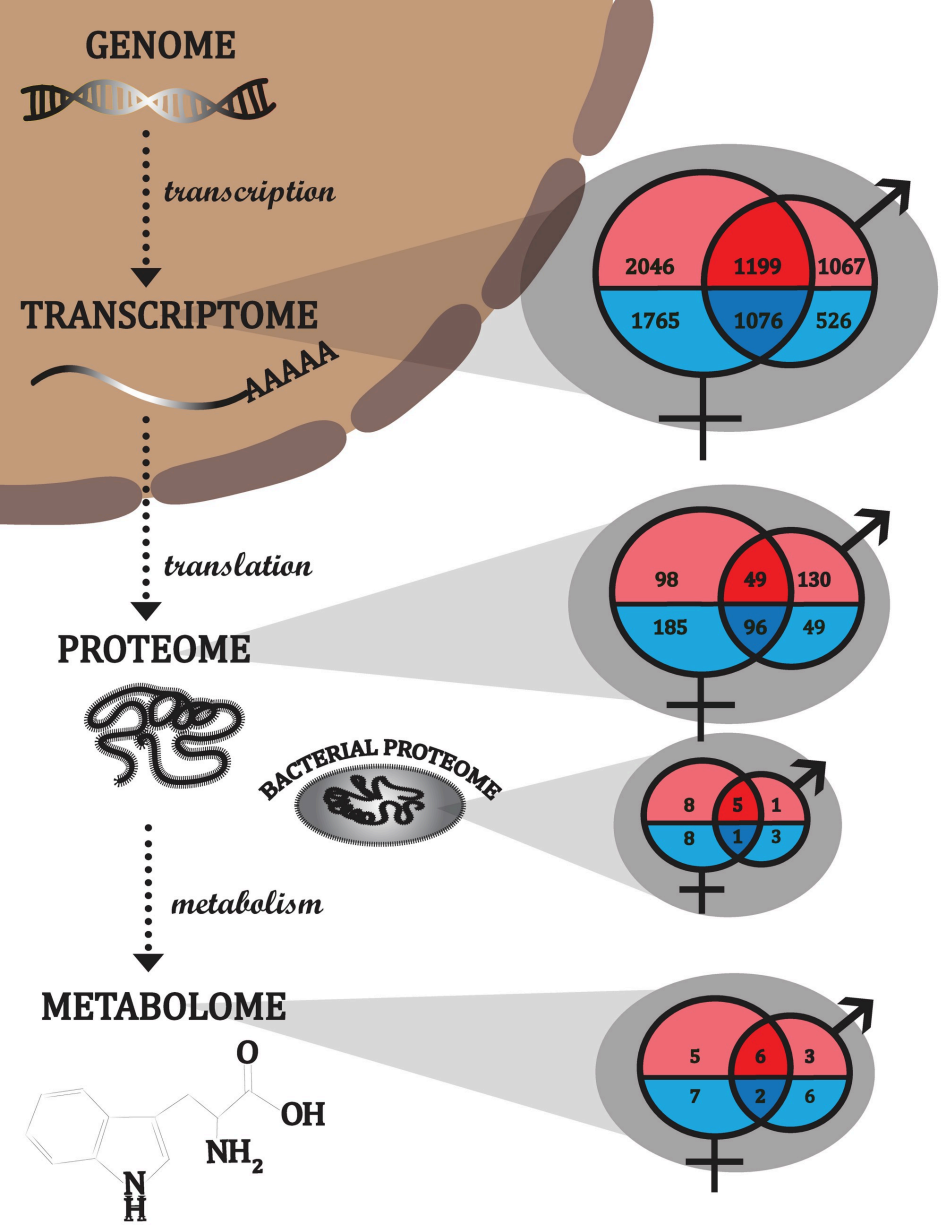
Numbers of differentially expressed genes at the level of the transcriptome (DEGs), differentially abundant proteins (DAPs), and differentially abundant metabolites (DAMs) in control vs. CLas-exposed males and control vs. CLas-exposed females. The female and male sex symbols are used as Venn diagrams to summarize the numbers of sex-specific and shared DEGs, DAPs and DAMs associated with CLas exposure. Those more expressed/abundant in CLas-exposed *D*. *citri* compared to controls are shown in red while those less expressed/abundant are shown in blue.

There was a more pronounced response to CLas exposure in females compared to males in terms of numbers of differentially expressed genes at the transcript level (6,088 DEGs in females versus 3,866 DEGs males) (Fig 7 and S2 and S3 Datasets), numbers of differentially abundant *D*. *citri* proteins (428 DAPs in females versus 324 DAPs in males) (Fig 7 and S4 and S5 Datasets), numbers of differentially abundant endosymbiont proteins (22 DAPs in females versus 10 DAPs in males) (Fig 7 and S4 and S5 Datasets), and numbers of differentially abundant metabolites (20 DAMs in females versus 17 DAMs in males) (Fig 7 and S1 Dataset). Additionally, males and females shared relatively few responses to CLas exposure among DEGs (29.6%), DAPs of *D*. *citri* (23.9%), DAPs of endosymbionts (23.1%), and DAMs (21.6%) (Fig 7). However, it is unlikely that these sex-specific responses to CLas exposure result in significant sex differences in susceptibility to CLas because we did not find a difference in CLas density or relative abundance between males and females (Figs 1 and 2).

### Male and female *D*. *citri* similarly alter regulation of cuticle- and chitin-containing barriers with CLas exposure

**Fig 8:**
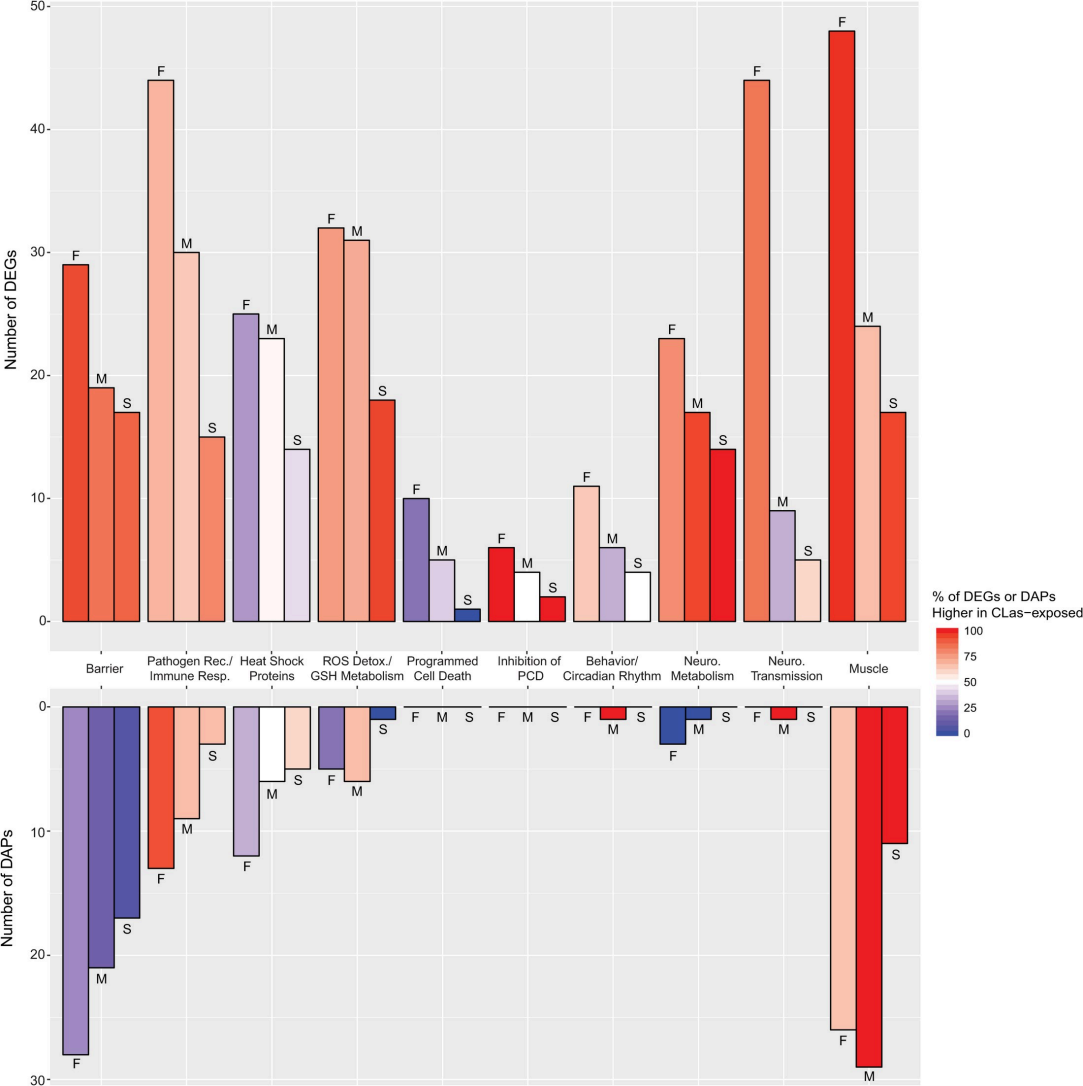
Number of DEGs and DAPs, and percentage that are more expressed/abundant with CLas exposure, by predicted function. The number of DEGs or DAPs for a particular functional category from females (bars marked with “F”), for males (bars marked with “M”), and shared between sexes (bars marked with “S”) in response to CLas exposure are depicted by the bar height. The percentage of DEGs or DAPs in that functional category that are up-regulated with CLas exposure for that group (females, males, or shared) is depicted by the color of the bars.

The gut epithelium of *D*. *citri* maintains the first critical barrier to CLas and other microbes ingested during feeding. The intima of the foregut and hindgut and the midgut peritrophic matrix (if present in *D*. *citri* [70]) incorporate cuticular proteins and chitin to create a protective barrier at the gut-microbe-food interface [71,72]. Similarly, cuticular proteins and chitin are essential components of the exoskeleton which shields *D*. *citri* from diverse biotic and abiotic factors in the environment. CLas-exposed males and females versus controls had greater expression at the transcript level of numerous genes involved in the integrity of these protective barriers (see “Barrier” Fig 8 and S6 Dataset). Despite the greater transcript-level expression of genes potentially involved in the exoskeleton, the abundance of exoskeleton- and peritrophic matrix-related proteins were lower, on average, in CLas-exposed *D*. *citri* compared to controls (Fig 8 and S7 Dataset). While the overwhelming majority of DEGs for cuticular proteins were more highly expressed in CLas-exposed males and females compared to controls, the vast majority of DAPs were less abundant. Genes encoding enzymes for chitin degradation to N-acetyl-D-glucosamine (e.g. chitinases, hexosaminidases) [71], or for chitin degradation into antibacterial chitosan [73] and ethanol (e.g. deacetylase domain-containing proteins, malic enzymes, alcohol dehydrogenase) [74] mostly had higher expression at the transcript level in CLas-exposed males and females compared to controls (S6 Dataset). In CLas-exposed males, chitinase and malic enzyme were also more abundant, but alcohol dehydrogenase was less abundant in both sexes and females had fewer malic enzymes (S7 Dataset). Lower abundance of many cuticular proteins in CLas-exposed males and females versus controls suggests the cuticle, and any existing peritrophic matrix in the midgut, have compromised integrity during CLas exposure. Since CLas must cross the gut barrier to reach the salivary glands and be transmitted, a weakened cuticle layer and/or peritrophic matrix along the alimentary canal may facilitate CLas invasion into epithelial cells and ultimately promote systemic infection. The cuticle is also critical in preventing fungal infections [75]; therefore any lessening of this barrier could benefit pathogenic fungi and might explain why CLas-exposed adult *D*. *citri* are more susceptible to fungal pathogens than unexposed *D*. *citri* [22,23].

### *D*. *citri* immune response to CLas exposure depends on sex

If the gut barrier is not sufficient to prevent CLas invasion, *D*. *citri* can potentially mount an immune response to encapsulate and neutralize CLas. It was found that CLas-exposed males and females compared to controls had greater transcript-level expression of genes and greater abundance of proteins involved in pathogen recognition and immune response, with most being altered in females (Fig 8 and S6 and S7 Datasets). Furthermore, few of these pathogen recognition and immune response genes and proteins were similarly altered in both sexes. Among putative pattern recognition receptors (PRRs), which are potentially involved in binding to pathogen-associated molecular patterns (PAMPs) or pathogen-activated ligands, 21 were more abundant at the transcript level in CLas-exposed females versus controls, while in CLas-exposed males only seven were more abundant compared to controls. Five PRRs were similarly altered at the transcript level among both sexes, including: greater expression of predicted C-type lectin mannose binding protein [76,77], E-selectin (which contains C-type lectin domains), sushi von Willebrand factor type A, EGF and pentraxin domain-containing protein 1 (which contains a C-type lectin domain and conserved domains of complement control repeats) and leucine-rich repeat transmembrane neuronal protein 2 [78]. The transcript-level gene expression of a predicted leucine-rich repeat containing protein 16A was lower in CLas-exposed males and females compared to controls. At the protein level, a vitellogenin [35] and apolipophorin [79] were more abundant in both CLas-exposed males and females versus controls (S7 Dataset). Elsewhere, *D*. *citri* leucine rich repeat (LRR) domain proteins [24,26], vitellogenins, and apolipophorins [25,27] were reported to be differentially abundant during CLas exposure. The heightened production of vitellogenin may explain the greater fecundity of CLas-infected *D*. *citri* females compared to control females [21]. Interestingly, the related and putative pathogen of Zebra chip, ‘*Candidatus* Liberibacter solanacearum’ (CLso) may cause reduced vitellogenin production in its female potato psyllid vector, *Bactericera cockerelli* [80] which may also explain reduced fecundity of CLso-infected females [80]. Unlike adult *D*. *citri* [7,8], *B*. *cockerelli* is able to acquire CLso during the adult stage relatively quickly with a majority of adults carrying CLso after just a three-day acquisition access period [81]. This provides further evidence suggesting that infection with Liberibacter causes changes in vitellogenin production which may directly impact susceptibility and fecundity. It should also be noted that vitellogenins are regulated by hormones, especially juvenile hormone and ecdysone, which are impacted by reproduction and nutrient status, and especially amino acid abundance [82,83]. Since this study did not separate virgin from mated females, we do not know if the proportions of mated and virgin females differed between control and CLas-exposed *D*. *citri*, and could potentially be a confounding factor in our findings on the impact of CLas exposure on female vitellogenesis. Moreover, CLas infection in the host plant is known to alter the nutritional composition of the phloem, including amino acids, and this in turn may have affected vitellogenesis [84–86]. Nonetheless, these shared PRRs remain good candidates to examine for recognition of, and defense against, CLas because there is evidence of their involvement in lipopolysaccharide recognition and immune response regulation [87,35,79].

Upon binding to PAMPs or other pathogen-activated ligands, PRRs can promote phagocytosis and initiate signaling cascades to trigger anti-microbial peptide production, apoptosis, inflammation, clotting, iron sequestration and melanization [88]. Canonical signaling cascades involved in mounting an immune response include the Toll, Immune Deficiency (Imd), c-Jun N-terminal kinase/mitogen-activated protein kinase (JNK/MAPK), and janus kinase/signal transducers and activators of transcription (JAK/STAT) pathways. The Toll pathway is particularly important in responding to fungi and Gram-positive bacteria, although in some Lepidopteran and Coleopteran insects, Gram-negative bacteria also activate the Toll signaling pathway [89,90]. In CLas-exposed *D*. *citri*, the Toll pathway may have been down-regulated at the transcript level as suggested by lower transcript-level gene expression of the leucine-rich repeat flightless-interacting protein 2 [91] and an uncharacterized protein homolog to myeloid differentiation primary response 88 (MyD88) [92] in CLas-exposed males, and the lower expression of serine/threonine-protein kinase pelle [93], evolutionarily conserved signaling intermediate in Toll pathway [94], TNF receptor-associated factor 6 (homologous to dTRAF2) [95], and cactin [96] in CLas-exposed females compared to controls. The Imd pathway recognizes Gram-negative bacteria; however, homologs of some participating proteins, including Imd, FADD, Dredd, IKKG, and Relish, have not been found in *D*. *citri* [68]. Regulation of the Imd pathways may be minimally altered in females, as suggested by greater transcript-level gene expression of the protein sickie, a necessary protein for Relish activation in *Drosophila* [97].

In *Drosophila*, activation of the Imd signaling cascade leads to activation of the JNK/MAPK pathway downstream of Tak1 [98]. In CLas-exposed males, E3 ubiquitin protein ligase SH3RF1 (also known as plenty of SH3s) [99], mitogen-activated protein kinase kinase kinase 13 (also known as mixed lineage kinase, and homologous to slipper in *Drosophila*) [100], and dual specificity mitogen-activated protein kinase kinase 7 (homologous to MKK hemipterous) [101] are all involved in JNK activation and had lower transcript-level gene expression compared to controls. Compared to males, females had a unique set of DEGs involved in JNK/MAPK activation, and regulation of the pathway appeared less congruent. Both males and females had lower transcript-level gene expression of dual specificity MKK hemipterous; however, TNF receptor-associated factor 4 (homologous to DTRAF1 and activator of the JNK cascade) [102] and JNK-interacting protein 1 (homologous to *Drosophila* APLIP1), a putative scaffolding protein and facilitator of JNK signaling [103], were more highly expressed at the transcript-level in CLas-exposed females versus controls.

Invading microbes can cause injury and stress which stimulate production of cytokines that initiate JAK/STAT signaling upon binding to the transmembrane receptor Domeless. Ligand-bound Domeless leads to association and activation of the cytoplasm-residing tyrosine kinase Hopscotch [104]. Subsequently, STAT proteins are recruited and phosphorylated by Hopscotch, and then translocate to the nucleus where they cause transcriptional activation of genes involved in the immune and stress response [104]. The JAK/STAT pathway may be further regulated by a suite of proteins including E3 SUMO-protein ligase PIAS3 which inhibits activated STAT [105]. While it remains unknown which cytokines in *D*. *citri* stimulate JAK/STAT signaling, it is noteworthy that only CLas-exposed females had greater transcript-level gene expression of pro-inflammatory cytokines, including pro-interluekin-16-like protein [106] and macrophage migration inhibitory factor homolog [107] compared to controls. Moreover, only CLas-exposed females presented evidence of altered JAK/STAT regulation in response to CLas exposure as indicated by lower transcript-level gene expression of Hopscotch and E3 SUMO-protein ligase PIAS3. Overall, the Toll, Imd, and JNK/MAPK signaling pathways were largely down-regulated at the transcript level for CLas-exposed adults, with very few genes differentially expressed at the transcript level in both sexes.

Although the signaling cascades, well-documented for their involvement in mounting a defensive response to bacterial pathogens and pathogen-induced stress, were not obviously up-regulated, there was still evidence of heightened host defenses in CLas-exposed males and females versus controls. CLas infection may have activated clotting and melanization in *D*. *citri* as indicated by greater transcript-level gene expression of the proclotting enzyme in CLas-exposed females, hemocyanins (automatically annotated as allergen Cr-PI-like or probable cyclin-dependent serine/threonine-protein kinase, but with conserved hemocyanin domains) in CLas-exposed males and females, and larger protein abundance of phenoloxidase subunit A3 in CLas-exposed males compared to controls. However, at the protein level, there was a lower abundance of two hemocyanins in males: XP_008477906.1 which may form homodimers in *D*. *citri*, and XP_008477908.1 which may form a heterodimer with the former [25]. Additionally, CLas-exposed females had a lower protein abundance of hemocyanin XP_008477908.1, but a greater protein abundance of hemocyanin XP_008477906.1 compared to controls. Of these differentially regulated hemocyanins, only XP_008477906.1 was predicted to have a signal peptide and was found interacting with a CLas protein [25]. This hemocyanin was also more abundant in mixed sex nymphs and adults when exposed to CLas [25]. Although this hemocyanin is impacted quite consistently by CLas exposure and directly interacts with CLas, when Hosseinzadeh et al knocked down its expression they unexpectedly found evidence of lower CLas density [108]. This highlights the importance of directly investigating potential anti-CLas *D*. *citri* responses to determine the impact on CLas transmission.

Proclotting enzyme causes coagulation and initiates phenoloxidase activity in the horseshoe crab with conversion of hemocyanin into phenoloxidase [109]. Hemocyanin is homologous to phenoloxidase, and in numerous other arthropods has demonstrated phenoloxidase activity in the presence of host- and microbe-derived factors [110]. Importantly, phenoloxidase activity is responsible for melanin-based encapsulation of microbial pathogens, and production of reactive oxygen species (ROS) innate immunity in insects [111]. Melanin synthesis involves the hydroxylation of phenylalanine to make tyrosine and the hydroxylation of tyrosine by tyrosine 3-monooxygenase to make L-DOPA. Phenoloxidase (whether in the form of hemocyanin or phenoloxidase subunit A3) may then oxidize L-DOPA to make dopaquinone and ultimately melanin. Aromatic L-amino acid decarboxylase (also known as Dopa decarboxylase) may also convert L-DOPA into dopamine which phenoloxidase can eventually convert into melanin. Interestingly, both phenylalanine and tyrosine were less abundant in CLas-exposed males, and tyrosine 3-monooxygenase and aromatic L-amino acid decarboxylase were expressed more highly at the transcript-level in CLas-exposed males and females compared to controls. Furthermore, there was evidence that adult *D*. *citri* defended against CLas propagation by limiting the availability of iron via greater transcript-level gene expression and protein abundance of ferritin and transferrin, particularly in CLas-exposed males compared to controls [112]. CLas-exposed males and females versus controls also had higher transcript-level expression of a gene for gamma-interferon-inducible lysosomal thiol reductase-like protein, a protein that reduces disulfide bonds in lysosomes, endosomes, and phagosomes [113], and is involved in inhibiting *E*. *coli* abundance in *D*. *melanogaster* [114]. Additionally, CLas-exposed females, versus controls, had greater transcript-level expression of genes with antimicrobial functions, including lysozymes which hydrolyze bacterial cell wall peptidoglycan [115] and dual oxidases which generate cytotoxic reactive oxygen species pivotal in controlling bacterial densities along the insect gut epithelial barrier [116]. Given that males and females did not have different densities of CLas in this study, their shared defensive responses might be particularly important in CLas infection.

### Evidence of cellular stress, particularly in CLas-exposed male *D*. *citri*

Numerous genes with predicted function as heat shock proteins were differentially regulated in CLas-exposed compared to control *D*. *citri* (Fig 8 and S6 Dataset). Most of those differentially regulated in females were lower at the transcript or protein levels in CLas-exposed females versus controls, but in males about half of DEGs and DAPs for heat shock proteins were lower and the other half greater in CLas-exposed males versus controls. Heat shock proteins are ubiquitous and conserved chaperones that aid in protein folding and refolding and modulate apoptotic processes [117]. They are especially up-regulated during physiologically stressful conditions such as extreme temperature, oxidative stress, and inflammation, to minimize cellular damage and apoptosis [118,119]. Greater transcript-level expression of genes involved in detoxification of ROS and metabolism of glutathione (a critical antioxidant in regulation of cellular redox state and thus apoptosis [120]), suggests that CLas exposure caused oxidative stress in *D*. *citri* (Fig 8 and S6 Dataset). In particular, glutaredoxins [121] and catalases [122] were consistently highly expressed in CLas-exposed *D*. *citri* compared to controls. Additionally, CLas-exposed males only exhibited an average greater abundance of ROS detoxifying enzymes (Fig 8 and S7 Dataset) and lower glutathione concentration (Fig 5 and S1 Dataset) compared to controls, indicating that they had elevated oxidative stress and perhaps more apoptosis than females due to CLas exposure.

Analysis of genes putatively involved in programmed cell death (PCD) (Fig 8) or inhibition of programmed cell death (Fig 8) suggests each sex regulates PCD uniquely in response to CLas exposure. CLas-exposed females mostly displayed lower transcript-level expression of genes involved in PCD and higher transcript-level expression of genes involved in inhibition of PCD. There was evidence that apoptosis, a form of PCD, was down-regulated in CLas-exposed females via the extrinsic and intrinsic pathways. Most DEGs for autophagy-related proteins were lower in CLas-exposed females versus controls. This includes Atg6 (“beclin-1-like protein”), Atg10 (“ubiquitin-like-conjugating enzyme ATG10”) and Atg101 (S6 Dataset). These proteins are necessary factors in autophagy [123,124], a form of programmed cell death that can occur in response to stress stimuli [125]. Additionally, genes involved in inducing apoptosis, caspase-1 [126] and programmed cell death protein 5 [127], had lower transcript-level expression in CLas-exposed females compared to controls. Most DEGs shown to inhibit or mitigate apoptosis, including lifeguard proteins 1, 2, and 4 [128], growth hormone-inducible transmembrane protein [129], and bax inhibitor 1 [130], were more highly expressed in CLas-exposed females versus controls. Like CLas-exposed females, CLas-exposed males too had lower transcript-level expression of Atg101 and anti-apoptotic lifeguard protein 1 and growth hormone-inducible transmembrane protein, compared to controls. However, unlike CLas-exposed females, CLas-exposed males had higher transcript-level gene expression of the potentially pro-apoptotic programmed cell death protein 10 [131], and lower transcript-level gene expression of anti-apoptotic bax inhibitor 1 and apoptosis inhibitor 5 homolog [132] compared to controls. Nonetheless, transcript-level gene expression of protein croquemort, a homolog of CD36 that is necessary for phagocytosis of apoptotic cells and enhanced upon increasing apoptotic cell numbers in *Drosophila* [133], was more highly expressed in CLas-exposed males and females versus controls.

ROS generated from host defense responses can cause oxidative stress in the host. There was considerable evidence of oxidative and cellular stress in CLas-exposed *D*. *citri*, especially CLas-exposed males, compared to controls and this is in accordance with findings reported by Mann and others in which only male adults, not females, had significantly more mitochondrial oxidative stress in the gut when reared on CLas-infected citrus [33]. Similar to our findings on the whole body of CLas-exposed males and females, mixed sex *D*. *citri* guts showed lower abundance of mitochondrial proteins when exposed to CLas [26]. However, a potential, alternate explanation for the increased oxidative stress is oral exposure to increased levels of H_2_O_2_ in citrus as a result of CLas infection in the plant [134].

### Altered proteins involved in behavior, neurobiology, and muscle

Compared to males, females had more DEGs involved in behavior and/or circadian rhythms due to CLas exposure, and few were similarly differentially expressed in males (Fig 8 and S6 Dataset). Notably, several *takeout* genes were differentially expressed at the transcript level in CLas-exposed males and females versus controls. Also, only CLas-exposed males had differential abundance of protein takeout compared to controls, and it was greater in CLas-exposed males (S7 Dataset). *Takeout* homologs are an evolutionarily conserved gene family in insects [135], first discovered in *Drosophila melanogaster* where its expression is regulated by the circadian clock and enhanced upon starvation [136]. It also modulates feeding [137], locomotion [136], and male courtship behavior [135].

Neurotransmitter abundance and neurotransmitter transmission are also critical to regulating behavior, and we found the transcription of genes involved in neurotransmitter metabolism to be greater in CLas-exposed males and females compared to controls (Fig 8). Many of these corresponding enzymes were not identified in the proteomics analysis. As mentioned previously, genes encoding enzymes in dopamine biosynthesis were more highly expressed at the transcript level in CLas-exposed male and female *D*. *citri* than controls, and phenylalanine and tyrosine, precursors to dopamine biosynthesis, were lower in CLas-exposed males than in controls. Dopamine, a neurotransmitter in animals, regulates a range of behaviors in insects such as social interactions, feeding, locomotion, and sleep, and also drives reward-seeking and aversive learning [138]. However, aromatic-L-amino-acid decarboxylase also catalyzes the conversion of 5-hydroxytryptophan to serotonin [139], and the precursor of 5-hydroxytryptophan, tryptophan, was more abundant in CLas-exposed *D*. *citri* than controls (Figs 5 and 6). Like dopamine, serotonin is a neurotransmitter that modulates a variety of behaviors and neurological processes, including feeding, sleep, circadian rhythms, locomotion, and memory [140]. Metabolism of the major excitatory neurotransmitter, glutamate, and major inhibitory neurotransmitter, 4-aminobutyrate (GABA) [141] may have also been altered. Transcript-level gene expression of 4-aminobutyrate aminotransferase and 5-oxprolinase, enzymes that catalyze the synthesis of glutamate from GABA and 2-oxoglutarate, and from 5-oxo-L-proline, respectively, was higher in CLas-exposed *D*. *citri* compared to controls. However, GABA was less abundant in CLas-exposed females compared to controls. Although there were many similarities in how CLas exposure impacted transcript-level regulation of neurotransmitter metabolism in males versus females, there were relatively few similarities in how CLas exposure impacted male versus female regulation of neurotransmitter transmission. A significant number of genes involved in neurotransmitter transmission via synaptic vesicular transport, neurotransmitter recognition, and neurotransmitter transmembrane transport were more expressed at the transcript level in CLas-exposed females versus controls, but were mostly less expressed in CLas-exposed males versus controls (Fig 8).

In males and females, CLas exposure was associated with greater transcript level expression of genes and greater abundance of proteins related to muscle structure (Fig 8 and S6 and S7 Datasets). Among *D*. *citri* proteins, there was evidence that the components of thick and thin filaments were differentially abundant in CLas-exposed *D*. *citri* than controls (S7 Dataset). This suggests that CLas-exposed *D*. *citri* had greater muscle mass than controls.

### Altered *D*. *citri* energy metabolism

**Fig 9:**
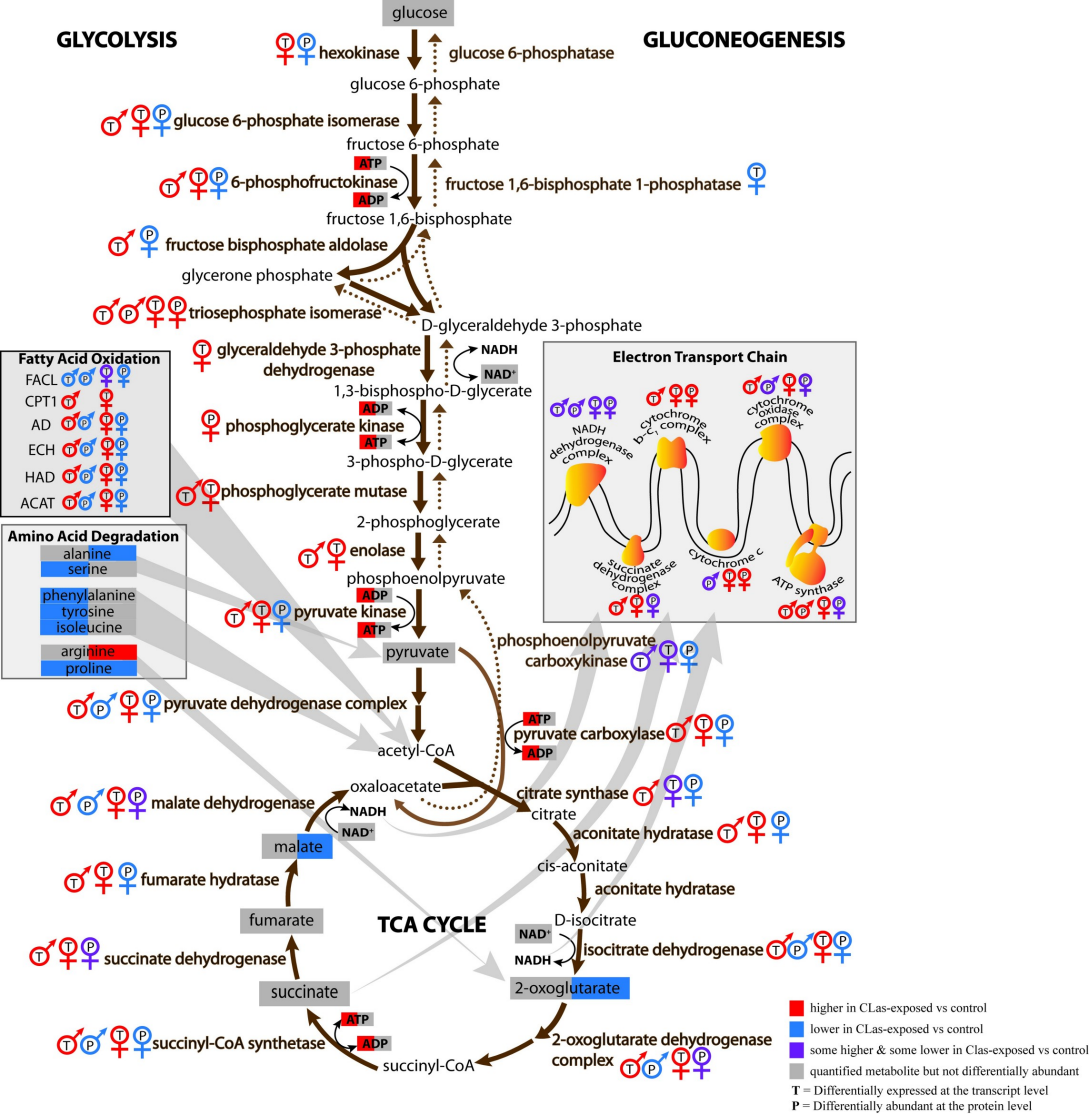
Pathway diagram of DEGs, DAPs and DAMs involved in glycolysis, gluconeogenesis, pyruvate decarboxylation, the TCA cycle, fatty acid oxidation, and the electron transport chain. DEGs (represented with a ‘T’), or DAPs (represented with a ‘P’) for males are depicted with the male sex symbol while those for females are depicted with the female sex symbol. Metabolites are highlighted in the left half if they were measured/differentially abundant in males, and are highlighted in the right half if they were measured/differentially abundant in females. Up-regulated DEGs/DAPs/DAMs are in red, down-regulated DEGs/DAPs/DAMs are in blue, measured but non-DAMs are highlighted in gray, and if some subunits or genes/proteins for the same enzyme are up-regulated and down-regulated then they are depicted in purple.

CLas exposure was associated with greater transcript-level expression of most genes involved in glycolysis, the tricarboxylic acid (TCA) cycle, fatty acid oxidation, and the electron transport chain in males and females (Fig 9 and S6 Dataset). Notably, there was greater transcript-level expression in CLas-exposed males and females of genes encoding enzymes for the rate-limiting steps of glycolysis (6-phosphofructokinase), TCA cycle (isocitrate dehydrogenase), and fatty acid oxidation (carnitine *O*-palmitoyltransferase). However, at the protein level, most enzymes involved in glycolysis, the TCA cycle, and fatty acid oxidation were lower in CLas-exposed males and females than controls (Fig 9 and S7 Dataset). In yeast mitochondria [142], there is relatively little correlation between mRNA and protein abundances and this may be due to post-transcriptional processing. Therefore, analysis of gene expression at the transcript and protein levels is particularly important to understanding perturbations to mitochondrial regulation.

ADP is phosphorylated to generate ATP in glycolysis, the TCA cycle, and by ATP synthase as a result of the proton gradient established by the electron transport chain. Both metabolites were quantified, and ADP and ATP were on average about 2.5-fold and 5-fold, respectively, greater in CLas-exposed males than controls (Figs 5 and 6, S1 Dataset). 2-Oxoglutarate and malate are essential metabolites in the TCA cycle and were 60% and 70% higher respectively, in control females compared to CLas-exposed females. Amino acids are also degraded to produce precursor metabolites for the TCA cycle. Among the differentially abundant amino acids that can be degraded for the TCA cycle, only proline was lower in CLas-exposed males and females versus controls (Fig 9). Other metabolites involved in energy metabolism including glucose, NAD+, pyruvate, succinate and fumarate were quantified but not significantly different in either CLas-exposed males or CLas-exposed females compared to controls.

Since CLas is hypothesized to scavenge ATP from its *D*. *citri* host [143], the changes in *D*. *citri* energy metabolism (reported here and reported previously [28,31]) while feeding on CLas-infected citrus could directly benefit CLas through increased generation of beneficial metabolites such as ATP. The simultaneous up-regulation of muscle proteins and takeout proteins, particularly in males, provides a possible connection between altered energy availability and increased dispersal and frequency of long-distance flights in CLas-infected, male *D*. *citri* [144]. Determining if CLas infection directly or indirectly alters *D*. *citri* flight behavior via regulation of energy metabolism, muscle or takeout proteins could inform approaches aimed at limiting dispersal of CLas-infected *D*. *citri*.

## Conclusions

In our novel investigation of male and female, and their microbial, responses to CLas exposure, we uncovered many changes in adult *D*. *citri* that were not known to occur similarly or differentially by *D*. *citri* sex at the transcript, protein, and metabolite levels. Since genomes, transcripts, proteins, and metabolites were isolated from the same unique *D*. *citri* samples, we provided the first systems-level molecular understanding of the *D*. *citri*-CLas-citrus relationship, and the importance of *D*. *citri* sex in these multitrophic interactions. Responses shared between males and females elucidated here, especially those related to *D*. *citri* defense and immunity, could be exploited to develop an intervention method that effectively prevents CLas transmission by both sexes in the field.

Importantly, we did not always find that CLas exposure impacted the *D*. *citri* transcriptome and proteome similarly, which echoes the lack of correlation between mRNA and protein levels found in other organisms and may be explained by post-transcriptional and post-translational processes [145]. This exemplifies the need to evaluate both the transcriptome and proteome in order to gain a more complete picture of how CLas exposure impacts its insect host. It must also be considered that these *D*. *citri* responses to CLas exposure may differ according to the citrus variety used for *D*. *citri* acquisition of CLas since others have reported variety-dependent expression of predicted CLas effectors [146] and variety-dependent impacts of CLas on the citrus leaf transcriptome [147] and proteome [148], and on the citrus root metabolome and microbiome [149]. Likewise, *D*. *citri*-CLas-citrus interactions may differ with *D*. *citri* population [150] and CLas strain [151]; therefore the HLB pathosystem should be studied under these differing variables to identify core *D*. *citri*-CLas-citrus interactions.

## Supporting information

S1 File Details on materials and methods used for extractions, PCR, qPCR, and ‘omics analysis. (DOCX)

S1 Table Chemical shifts and coupling constants of diaphorin in 90% H_2_O and 10% D_2_O. (XLSX)

S1 Fig Correlation of CLas density with endosymbiont densities. No correlation was found between: CLas density and Profftella density in males (squares) or females (triangles) (A); CLas and Carsonella densities in males (B); or CLas and *Wolbachia* densities in males (C). CLas, Profftella, and Carsonella densities were measured by the number of *16S rRNA* sequences to *D*. *citri Rps20* sequences, and log_10_-transformed. *Wolbachia* density was measured by the number of *Wolbachia ftsZ* sequences to *D*. *citri Rps20* sequences, and log_10_-transformed.
(PDF)

S2 Fig Correlation of CLas density with diaphorin concentration. No correlation was found between CLas density and diaphorin concentration in males (squares) or females (triangles). CLas density was measured by the number of CLas *16S rRNA* sequences to *D*. *citri Rps20* sequences, and log_10_-transformed. Diaphorin concentration (μmol/g *D*. *citri*) was log_10_-transformed.
(PDF)

S1 Dataset Mean metabolite concentrations. (XLSX)

S2 Dataset Differentially expressed genes between control and CLas-exposed adult male *D*. *citri*. (XLSX)

S3 Dataset Differentially expressed genes between control and CLas-exposed adult female *D*. *citri*. (XLSX)

S4 Dataset Differentially abundant proteins between control and CLas-exposed adult male *D*. *citri*. (XLSX)

S5 Dataset Differentially abundant proteins between control and CLas-exposed adult female *D*. *citri*. (XLSX)

S6 Dataset Differentially expressed genes and corresponding predicted functions used to generate Figs 8 and 9. (XLSX)

S7 Dataset Differentially abundant proteins and corresponding predicted functions used to generate Figs 8 and 9. (XLSX)
