# Supplementary material for: Development on Citrus medica infected with ‘Candidatus Liberibacter asiaticus’ has sex-specific and -nonspecific impacts on adult Diaphorina citri and its endosymbionts
Source: PLoS One. 2020 Oct 6;15(10):e0239771. doi: 10.1371/journal.pone.0239771 (PMC7537882; doi:10.1371/journal.pone.0239771)
Supplement: S1 File — (DOCX) [file pone.0239771.s001.docx]

**Supplemental Materials and Methods**

***Metabolome analysis***

For each PCR, 4 µL of 5X KAPA HiFi buffer was combined with 3.6 µL PCR-grade water, 0.6 µL of 10 µM dNTP mix, 0.4 µL of KAPA HiFi Hot Start Polymerase, 1 µL of DMSO, 2 µL of 2 µM forward primer, 0.4 µL of 10 µM reverse primer, and 8 µL of DNA template. PCR conditions included initial denaturation at 95 °C for 5 min, 25 cycles of denaturation at 98 °C for 20 s, annealing at 55 °C for 15 s, and elongation at 72 °C for 60 s, followed by a final extension at 72 °C for 10 min. PCR products were analyzed by gel electrophoresis and were quantified by comparing to the BioRad EZ Load 100 bp ladder intensity. Equal amounts of PCR product from each sample were combined, and cleaned using the QIAquick PCR Purification kit (Qiagen) following the manufacturer’s instructions.

***Metabolite, DNA, RNA and protein extraction***

Prior to extraction, four 2 mm diameter zirconium oxide yttria stabilized beads (Glen Mills, Inc.), and about 130 mg of 0.1 mm diameter zirconia/silica beads (BioSpec) were cooled in liquid nitrogen and added into each sample, and all samples were cooled in liquid nitrogen for two minutes. The samples were then treated with three rounds of grinding in a Mini Beadbeater (BioSpec) for 20 sec followed by flash-freezing in liquid nitrogen for 30 sec. To each ground sample, 150 µL HPLC-grade methanol (> 99.9 % pure, cooled to -20 °C), 150 µL ultrapure water (cooled to -20 °C), and 300 µL HPLC-grade amylene-stabilized chloroform (> 99.9 % pure, cooled to -20 °C) were added. They were vortexed, bead beaten for 20 sec, and centrifuged for 5 min at 14,000 x g and 4 °C. Using a pipette, the top layer (polar metabolites) and bottom layer (nonpolar metabolites) were separated from the interphase layer and beads, and stored at -75 °C. The interphase layer was further processed with addition of 1050 µL of Qiagen buffer RLT (with β-mercaptoethanol added, cooled to 4 °C), vortexing, and bead beating for 30 sec. The lysate was then split evenly over two Qiagen QIAshredder columns, vortexed for 2 min at 16,100 x g, and combined before storing at -75 °C. Lysates were thawed in a water bath at 35 - 37 °C for 15 min, then using the Qiagen AllPrep DNA/RNA/Protein Mini Kit and following manufacturer’s instructions for “Simultaneous Purification of Genomic DNA, Total RNA, and Total Protein from Animal and Human Cells”, they were split evenly over three DNA columns. The following modifications/options were chosen in the instructions: at step 5, DNA on the column was stored at 4 °C awaiting washing; at step 6, 250 µL of 100 % ethanol was added to each flow-through (i.e. three per sample); instead of step 8, steps E1 through E4 on page 52 were followed for on-column DNase I treatment; the optional step 11 was performed; at step 12, RNA was eluted with 50 µL RNase-free water; dried protein pellets were not suspended in ALO buffer. All three elutes from one sample were separately combined for metabolites, DNA, RNA and proteins. RNA was further cleaned using the Zymo Research RNA Clean and Concentrator-5 Kit with DNase I. Following the manufacturer’s instructions, RNA was treated with DNase I, then cleaned.

***qPCR and thermal cycler conditions***

For CLas density from individual *D. citri*, 12.5 µL of GoTaq qPCR master mix was combined with 2 µL of 5 µM forward primer, 2 µL of 5 µM reverse primer, 3.5 µL water, and 5 µL gDNA. Quantitative PCR was carried out on the LightCycler 96 Real-Time PCR System (Roche) with DNA denaturation and polymerase activation at 95 °C for 500 s and 50 cycles of denaturation at 94 °C for 15 sec, annealing at 60 °C for 30 s, and extension at 72 °C for 30 s. Melt-curve analysis ensued and involved 60 s at 95 °C, 60 s at 40 °C, 30 s at 60 °C and 1 s at 90 °C.

For endosymbiont densities from samples containing 75 *D. citri*, 10 µL of SsoAdvanced Universal SYBR Green Supermix (BioRad) was combined with 0.8 µL of 10 µM forward primer, 0.8 µL of 10 µM reverse primer, 5 µL gDNA, and 3.4 µL PCR-grade water. Using the BioRad CFX96 Touch Real-Time PCR Detection System, polymerase chain reactions began with polymerase activation and DNA denaturation at 98 °C for 3 min, and continued with 40 cycles of denaturation at 98 °C for 15 s and annealing/extension/plate reading at 60 °C for 40 s. Melt-curve analysis was performed after each qPCR to confirm amplification specificity and involved 0.5 °C increases every 5 s starting at 65 °C and ending at 95 °C with a plate read after each temperature increase.

Standard curves for Liberibacter *16S rRNA*, Carsonella *16S rRNA*, *D. citri* *RPS20*, Profftella *16S rRNA*, and *Wolbachia ftsZ* were generated using ten-fold dilutions of plasmid containing gene sequence in the vector pUC57 (GenScript).

***Gene expression***

Scythe version 0.991 was used to remove adapters from reads (prior contamination rate of 0.3; 5 bases as smallest length adapter to consider; filter sequences less than 35 bases), and reads were trimmed and filtered with Sickle version 1.33 (quality threshold of 10; 20 bases as length threshold; no 5’ trimming; truncation at first N position). HISAT2 alignment parameters included --max-intronlen 136000, --rna-strandness RF, --dta, --fr, --no-mixed, --no-discordant. Sorted BAM files were used to generate gene count tables with StringTie version 1.3.3 (using options -B, -e, --rf, and -A).

***Mass spectrometry-based quantitative proteomics***

Dried protein pellets were washed with acetone, dried, and then resuspended in a solution containing 8 M urea and 50 mM triethylammonium bicarbonate in water, and then centrifuged. The resulting supernatant was used for protein quantification, quality assessment, and LC/MS/MS analysis. Using Mascot Daemon v2.5.1, spectra were mapped to predicted tryptic peptides in a database containing predicted proteins from the *D. citri* genome assembly v1.1, *D. citri* MCOT v1.0 transcriptome, Carsonella genome assembly, Profftella genome assembly, *Wolbachia* genome assembly, and CLas genome assembly for strain psy62. Using Scaffold v4.8.4, peptide spectra were mapped to proteins in the database and the normalized spectral count was calculated for each protein and for each replicate biological sample
