# Supplementary figures and images for: Development on Citrus medica infected with ‘Candidatus Liberibacter asiaticus’ has sex-specific and -nonspecific impacts on adult Diaphorina citri and its endosymbionts
Source: PLoS One. 2020 Oct 6;15(10):e0239771. doi: 10.1371/journal.pone.0239771 (PMC7537882; doi:10.1371/journal.pone.0239771)

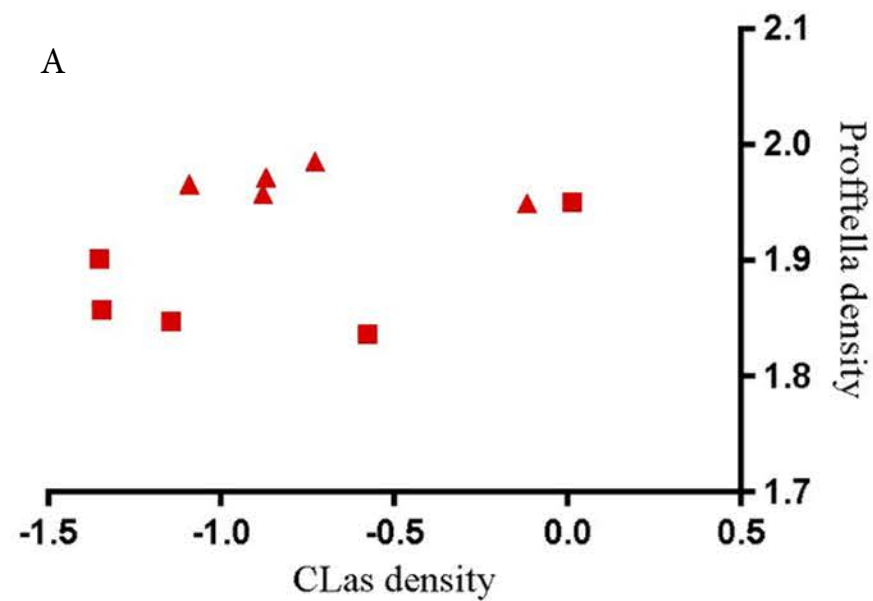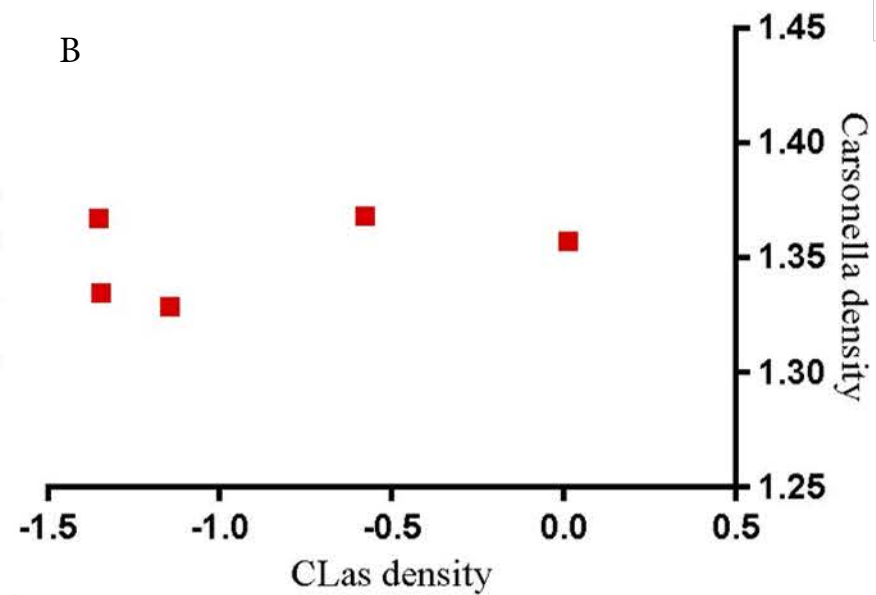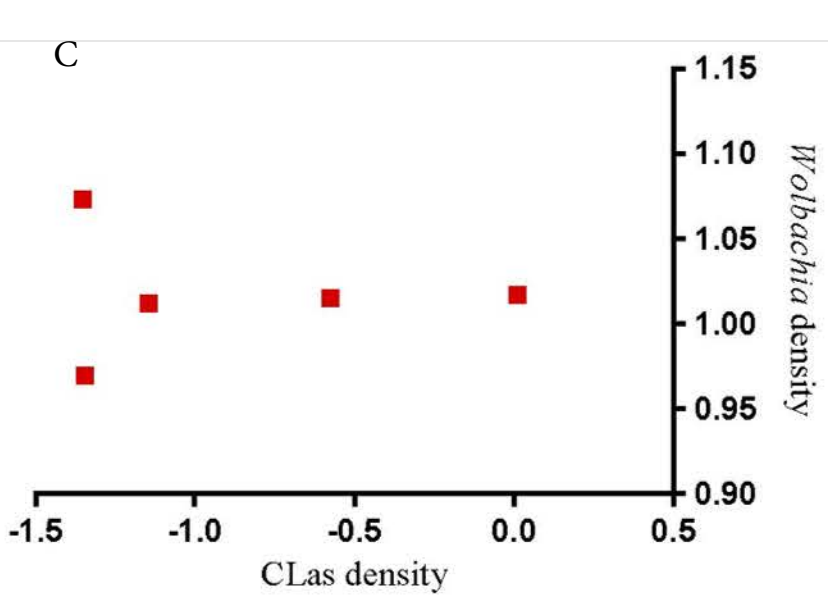

Supplement: S1 Fig — No correlation was found between: CLas density and Profftella density in males (squares) or females (triangles) (A); CLas and Carsonella densities in males (B); or CLas and Wolbachia densities in males (C). CLas, Profftella, and Carsonella densities were measured by the number of 16S rRNA sequences to D. citri Rps20 sequences, and log10-transformed. Wolbachia density was measured by the number of Wolbachia ftsZ sequences to D. citri Rps20 sequences, and log10-transformed. (PDF) [file pone.0239771.s003.pdf]

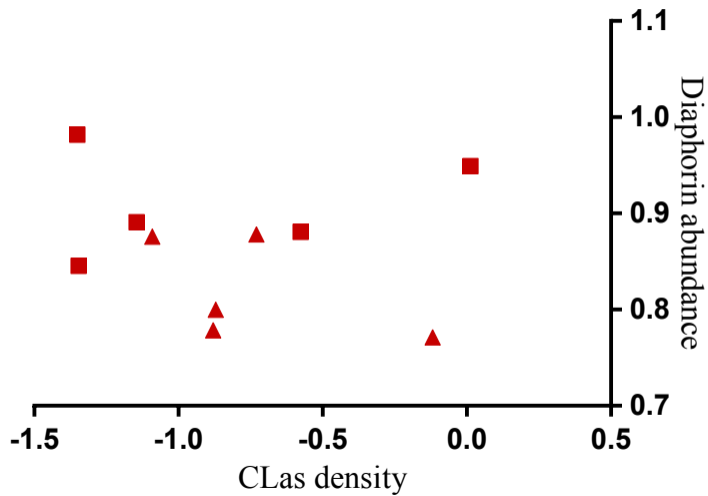

Supplement: S2 Fig — No correlation was found between CLas density and diaphorin concentration in males (squares) or females (triangles). CLas density was measured by the number of CLas 16S rRNA sequences to D. citri Rps20 sequences, and log10-transformed. Diaphorin concentration (μmol/g D. citri) was log10-transformed. (PDF) [file pone.0239771.s004.pdf]
